# A Case of Hydrocephalus Acutus

**Published:** 1817-06

**Authors:** Peter Fairbairn

**Affiliations:** Surgeon, R. N.


### *A Case of Hydrocephalus Acutus.* Communicated by Mr. Peter Fairbairn, Surgeon, R. N.

THE following is a fatal case, occurring within my observation, the peculiar features of which may not be unacceptable to some of the readers of the Medico-Chirurgical Journal; at all events, the appearances on dissection may perhaps tend to throw more and more light on the pathology of this insidious disease.—About two months ago, the patient, a boy of four years old, stout, healthy, and active, appeared dull and languid, as if fatigued by some unusual exertion; at intervals, he became lively, and fond of his former amusements. Countenance occasionally pale, and of a sallow hue; features contracted; eyes heavy, with a dark line under each eye-lid. Pulse natural; skin of a dry and harsh feel, with a slight increase of heat on its surface during night, terminating in partial sweats. Tongue clean; bowels unusually torpid; abdomen somewhat swollen, with a tenderness on pressure over the epigastrium; thirst moderate; appetite variable; sleep indifferent.

The child remained in this situation for upwards of six weeks, during which period there was little variation in the symptoms: at one time lively and playful; at another, languid, inactive, and peevish.

Purgative medicines of different kinds were administered, viz. calomel combined with jalap, rhubarb, magnesia, &c. These, although occasionally repeated, procured but scanty stools, in general of a firm consistence and of a clayish appearance.

On the morning of the 24th of February, he was attacked with symptoms of pyrexia, lassitude, some degree of vertigo, head-ache, pains in his limbs, eyes suffused and dull, pupils rather dilated, occasional flushes in the cheeks; pulse 95, of moderate strength; heat of skin not much increased; tongue covered with a whitish fur; bowels obstinately torpid; appetite greatly impaired. At times, the child started from his sleep, complaining of head-ache and pain over the abdomen.

From several unavoidable circumstances, I was prevented from seeing the child for the two subsequent days; therefore the symptoms during this period cannot, with certainty, be ascertained: however, I well know that an emetic of antimonial wine was given; after which, powders composed of calomel and jalap.

February 26th, three, P. M. Found him labouring under symptoms of oppressed brain; head-ache; vertigo; eyes suffused; pupils dilated; iris sensible to the stimulus of light. Pulse 100, rather full; skin hotter than natural; thirst inconsiderable; tongue furred; bowels much constipated. Applicent. hirud. No. vi. temporibus. Capt. ol. ricin. ℥iv. statim, et si alvus non. solvat. injiciat. enem. domestic.

27th. Slept well in the fore part of the night, but became restless in the morning. Leeches discharged freely. Head-ache and other symptoms relieved; iris perfectly sensible; pulse soft and less frequent; thirst moderate; skin at present cool. Had two very offensive dark greenish stools of a thin consistence after the injection.

℞. Submur. hydrarg. gr. ij; pulv. jalap, gr. v. M. ft. pulv. Capt. unum secund. quaq. hor. Injiciat. enem. si opus sit. Pediluvium vespere.

28th. Slept well; complains only of transient pain over the abdomen. Sat up in bed for some time, and conversed with his attendants. Pulse 95, of moderate strength; skin cool; tongue cleaner. Has taken eight powders, and one injection has been administered; after which, two very offensive dark greenish stools were procured. Has taken a little arrow-root. Omit. pulv.

Capt ol. ricin. ℥iv, et injiciat. enem. domest. si opust sit. Pediluvium vespere.

March 1st. Good night; this morning appears lively. Pupils observed to be somewhat dilated; in other respects the eye is of its natural appearance. Occasional pain is felt in the belly, which is removed by warm fomentations. Pulse increased in frequency; skin and thirst nearly natural.

℞. Submur. hydrarg. gr. jß; pulv. rhei gr. iij; magnes. gr. vj. M. ft. pulv. Capt. un. secund. quaq. hor. et injiciat enem. si opus sit. Pediluvium.

2d. Sleep interrupted by startings; pupils dilated, iris sensible. Has taken three powders, the last of which induced vomiting. Two injections have been given, which procured two loose bilious stools, in one of which a round worm was observed. Pulse frequent and rather weak; tongue pretty clean; skin and thirst nearly natural.

Omitt. pulv.; capt. ol. ricin. ℥ij, et enem. domest. si opus sit. Pediluv. vespere.

3d. Restless night. This morning is troubled with hiccough, and a short dry cough. Pain in the right hypochondriac and epigastric regions, increased on pressure or lying on the left side. Had one loose offensive stool of a viscid mucous appearance. Pupils dilated; iris sensible. Pulse 100, weak and irregular. Face occasionally flushed. Drank a little beef tea.

Capt. submur. hydr. gr. ij, tert. q. h.; applic. emplast. lyttæ later. dextr.; injiciat. enem. si opus sit.

4th. Symptoms increased; restless night; frequent startings. Features collapsed. Pupils dilated; iris insensible. Had two copious loose greenish stools. Pulse 110, weak and irregular. Face and breast bedewed with a cold sweat. — Rep. submur. hydr. gr. ij ut heri.

Became comatose after the morning visit, and remained in this situation till three P. M. when he was attacked with convulsions, which continued, with little intermission, till five o'clock; he then sunk into a comatose state, and expired about half past five P. M.

*Appearances on dissection.*—The cavity of the abdomen being exposed, no morbid or unnatural appearance in any of its viscera could, on the minutest inspection, be discovered.

On examining the chest, I was surprised to see the right lung discoloured, and apparently in a diseased state. I found a number of tubercles in each lobe, and several of the bronchial glands enlarged, two of which were fully as big as a pigeon's egg; and, on a section being made, presented the usual appearance of scrophulous structure. The lung being raised, about six ounces of serous fluid, tinged with blood, occupied that cavity, and marks of inflammation were seen in the pleura.

I next examined the brain, where I found an accumulation of fluid in the lateral ventricles, amounting, as nearly as I could guess, to three ounces: its membranes did not exhibit any very unusual vascular turgescence.

### Remarks.

In the foregoing case there is some reason for supposing that the fatal effusion had taken place previous to the 26th of February; at least, no great degree of vascular excitement was apparent after that time. In the detail of the symptoms there appear several peculiarities. The pupil, during the course of the disease, was observed more or less dilated; and, what is singular, the iris continued sensible till within a few hours of his death.

Nausea and vomiting, symptoms which, I believe, are present in almost every instance, were absent in this particular case. Although, in the report of the 2d of March, vomiting has been mentioned, yet, as it happened immediately after medicine had been given, I cannot conceive that it arose from any sympathy existing between the brain and stomach.

The above case shows the necessity of an early attention to the symptoms and progress of this very insidious and often fatal disease.

March 14th, 1817.

